# Allocating structure to function: the strong links between neuroplasticity and natural selection

**DOI:** 10.3389/fnhum.2013.00918

**Published:** 2014-01-07

**Authors:** Michael L. Anderson, Barbara L. Finlay

**Affiliations:** ^1^Department of Psychology, Franklin & Marshall CollegeLancaster, PA, USA; ^2^Neuroscience and Cognitive Science Program, Institute for Advanced Computer Studies, University of MarylandCollege Park, MD, USA; ^3^Behavioral and Evolutionary Neuroscience Group, Department of Psychology, Cornell UniversityIthaca, NY, USA

**Keywords:** cortex, modularity, evo-devo, visual system, neural re-use

## Abstract

A central question in brain evolution is how species-typical behaviors, and the neural function-structure mappings supporting them, can be acquired and inherited. Advocates of brain modularity, in its different incarnations across scientific subfields, argue that natural selection must target domain-dedicated, separately modifiable neural subsystems, resulting in genetically-specified functional modules. In such modular systems, specification of neuron number and functional connectivity are necessarily linked. Mounting evidence, however, from allometric, developmental, comparative, systems-physiological, neuroimaging and neurological studies suggests that brain elements are used and reused in multiple functional systems. This variable allocation can be seen in short-term neuromodulation, in neuroplasticity over the lifespan and in response to damage. We argue that the same processes are evident in brain evolution. Natural selection must preserve behavioral functions that may co-locate in variable amounts with other functions. In genetics, the uses and problems of pleiotropy, the re-use of genes in multiple networks have been much discussed, but this issue has been sidestepped in neural systems by the invocation of modules. Here we highlight the interaction between evolutionary and developmental mechanisms to produce distributed and overlapping functional architectures in the brain. These adaptive mechanisms must be robust to perturbations that might disrupt critical information processing and action selection, but must also recognize useful new sources of information arising from internal genetic or environmental variability, when those appear. These contrasting properties of “robustness” and “evolvability” have been discussed for the basic organization of body plan and fundamental cell physiology. Here we extend them to the evolution and development, “evo-devo,” of brain structure.

Brain evolution is an ultimate expression of neuroplasticity. Neuroplasticity, in turn, should inform us about what brain architectures have been selected over evolutionary time. If any current computer users were informed that their personal computers, which heretofore had been used only for word processing, could also store and transform images, few would be amazed. If the same people, however, were informed that their parents' video cameras, by simply adding new input and output devices, could function as word processors, they would probably be incredulous, and undertake a re-analysis of their presumptions about video camera technology. In the same way, understanding of how brains can change and understanding of neural architecture should bootstrap each other. The multiple kinds of brain plasticity—evolutionary, developmental, damage-induced and normal individuation—should be joined together into a natural unit for this investigation.

To understand how brains evolve, a central goal must be to distinguish a brain modification resulting from a direct genetic change in a single brain location from the spreading, downstream reorganization produced by adaptive nature of the brain itself responding to that genetic change. For example, a genetic change might directly cause the enlargement of the precursor pool for a single brain region and increase its neuron numbers, or, cause those neurons to express a new neurotransmitter, or, increase their axonal branching. Following on any one of these changes, however, the regions to which a genetically-altered region connects might be reconfirmed in any number of ways through “standing” mechanisms of neural development and plasticity, about which we now have extensive knowledge. For example, loss or gain in neuron numbers in connecting structures could result via changes in trophic support in normal developmental cell death, or changes in the volume of axonal and dendritic arbors via new activity levels, or new connectivity at the neuron level via Hebbian synaptic sorting under changed parameters. These mechanisms are the environment in which genetic change must operate. Although neuroplasticity was first understood through observations of adaptive responses to damage or stress, for example, rerouting of axons to new targets when a target was lost, or upregulation of excitability in case of denervation, it can now be understood in a larger context. The developing domain of “evo-devo,” the study of the selection of developmental mechanisms in evolution (Kirschner and Gerhart, 2005), is the context to understand neuroplasticity more broadly as stabilized adaptive responses to genetic as well as environmental variation. A few uncontroversial examples of how selection might favor some classes of developmental mechanisms over others will illustrate this idea. Co-regulation of neurogenesis, gliogenesis and vascularization rather than independent specification of each could make an entire such assembly more robust to variation in any part. The existence of basic Hebbian circuity or directed re-use of existing circuitry could enable either new environmental sources of information, or new information introduced by genetic change (e.g., new receptor sensitivities) to be automatically employed without requiring committed recognition circuitry to be generated by random variation and selected at each processing stage in the brain, allowing evolvability. No impermissible precognition resides in such mechanisms, only the fact that the normal opportunities, variations and disasters of life on earth, small and large, external and external, will progressively filter all organisms for those containing the suite of mechanisms that allowed their ancestors' survival.

Before we discuss these aspects of neuroplasticity, we need to examine assumptions about basic brain architecture that have proliferated independently in the different groups of scientists who concern themselves with the brain. Developmental biologists, neuroethologists, geneticists, anthropologists, psychologists, neuroscientists and cognitive and computer scientists each bring their own explanatory taxonomies to the investigation, each grappling with the relationship of their taxonomy to the physical parts of the brain using the analytical tools and measurements each has at hand. In this review, we will examine the interaction of particular concepts of brain architecture with mechanisms of neural development and plasticity. Particularly, we will discuss the idea of a “module,” a hypothesis about the relationship of species-typical behaviors (from escape behaviors in molluscs to language in humans) to single neurons or brain part. This hypothesis about function-structure relationships has different names and forms in different disciplines, such as “proper mass” or “mosaic evolution” in paleontology and comparative neuroanatomy, or “massive modularity” in psychology and cognitive science. Note that the sense of the word “module” as it is often used in neurobiology (e.g., Buxhoeveden and Casanova, 2002) to mean simply a unit or segment that can be iterated, like a cortical column, is not the sense of the term we are considering here. Our second focus is “evolvability” (Kirschner and Gerhart, 2005). Central to evo-devo is the resolution of the apparent paradox that existing developmental processes must have been selected both to be robust to perturbations and accidents, but not so robust as to not be “evolvable,” and should be able to allow, or even facilitate useful evolutionary variation. The concepts of “module” and “evolvability” have found extensive empirical intersection in the general problem of how to allocate neuron number, volume, and metabolic energy to important brain functions in each species independently, and across species. Here we will offer some initial proposals regarding how conserved developmental mechanisms may channel neural reuse, and begin the process of identifying those neural mechanisms that must eventually resolve the brain's evo-devo paradox, shedding light on how the allocation and reallocation of neural resources is made on both individual and evolutionary scales.

In the immediately following historical overview, we will concentrate on work that has described resource assignment and allocation in the brain. In anthropology, comparative neuroanatomy and neuroscience, researchers generally address neural number and brain volume, and integration of information at the level of single neurons. In current neuroimaging and cognitive neuroscience, volume and amount of activation, as determined in current neuroimaging methods, are the central measures. These brief histories are not meant to be comprehensive, but to remind readers of several intellectual threads we hope to integrate. We will interleave some empirical work on the developmental specification and control of neuron number, brain volume and brain activation with discussion of brain architecture and with this history, but will follow with more detailed examples after candidate brain architectures as they are understood in several disciplines have been laid out.

## The current status of the modularity debate

### Neuroscience and neuroethology

The reverse-engineering stance of the first electrophysiologists like Hubel and Wiesel (1962), Mountcastle et al. (1975) and Schiller et al. (1976), that much could be learned from investigating the responses of single neurons and inferring from their properties the mechanisms of perception and action, dominated the early years of central nervous system investigation. For these explicitly atheoretical, inductive approaches, the fact that single neuron responses in the cortex were reasonably interpretable by presenting simple stimuli to cats and monkeys produced an explosion of descriptive and systematic research. In due course these lines of work became subsumed under more analytically-driven work, such as the “single neuron doctrine” of Barlow (1972), and later by the more functionally driven “levels of analysis” approach of Marr (1982).

The idea of the brain as an evolved organ, however, was singularly absent from the work of all of these researchers. The idea that the brain is best viewed as a collection of functionally-committed circuits, each put in place by natural selection, rose in parallel, in opposition to the initial descriptive approach (Camhi, 1984). Neuroethologists concatenated a number of strategies and hypotheses together in their evolutionary approach: that the best kinds of behavior for study were those essential for survival, under strong, species-typical selection (e.g., mating calls or other species-typical communication, or prey capture and recognition); that these were best studied in “simpler” systems than large mammals; and that attention must be paid to the environment of each species and how every signal should be perceived or produced in relation to that context. Finally, it was assumed the nervous system ideally should be specialized for these specific adaptive ends throughout, from receptor to motor neuron. In essence, each piece of adaptive behavior was imagined to be supported by a committed, specialized module.

In the famous case of frog prey capture, the center-surround receptive fields were characterized as “fly detectors” (Lettvin et al., 1959); in the toad, “worm” and “anti-worm” signaling at the level of the midbrain tectum was said to direct prey-strike or avoidance directly (Ewert, 1984). The frog auditory system bracketed in its two auditory end-organs the two essential croak frequencies, and the hunt was on to locate the and-gate for those two frequencies, a single-neuron “croak detector” somewhere in the frog brain (Frishkopf et al., 1968). For songbirds, the “song system” was designated within the bird brain and each component given its own song-specific nomenclature (Nottebohm et al., 1976). (This initial description virtually precluded understanding song in terms of the non-song circuitry it was embedded in and presumably derived from, now being redressed—e.g., Goldberg et al., 2012). In invertebrates, the useful feature of individually-identifiable, large neurons raised the prospect of a direct map of the circuitry of these large neurons to a short list of the behaviors these animals could produce (Camhi, 1984). As with reverse engineering, the neuroethological approach yielded an explosion of useful information. In due course, in virtually every case, realization began to arise that not every feature of adaptive behavior mapped directly to corresponding special adaptations in neurons and neuronal circuits, and that many features were vertebrate- (and invertebrate-) general. Some of these findings will be listed here, and we'll return to a more specific discussion of aspects of neuroplasticity in this domain later.

Across the board, it became apparent that peripheral receptors (at least in vertebrates) are rarely tuned narrowly to the frequency of a communication channel, or the color of a preferred food, but rather tend to be broadly tuned to extract information in the channel of interest, the entire visible spectrum, for example (Lythgoe and Partridge, 1989; Kocher, 2004; Spady et al., 2006; Osorio and Vorobyev, 2008). Often, it appeared that it was the signal rather than signal decoding that had undergone selection to be maximally apparent or attention-getting to a generic nervous system, termed “sensory exploitation.” Systems as diverse as visual signaling in fish and anoles, mating croaks in frogs, and fruit identification in primates have this characteristic (Ryan, 1998; Persons et al., 1999). Specializations nested in broad channels, such as visual and auditory “foveas” are more common adaptations than commitment of all resources to a specialized bandwidth. In parallel, the computational complexity of the problems common to all vertebrates (or, in fact, all mobile life-forms) began to be better appreciated. General functions such as localization of items of interest in egocentric space, construction of topographic maps, learning the benefits and consequences of particular cues or environments, recognizing food or conspecifics as a category (as distinguished from recognition of one's own species), and motor control, all had to co-exist with any specialized circuitry. Generic “environments” exist as well: across niches, visual and acoustic environments on earth, “natural scenes,” proved to have a specific, statistical structure that all nervous systems of any complexity must exploit (Field, 1994; Lewicki, 2002).

Even for the simplest system, considering cases of invertebrates with small numbers of neurons, and smaller-still numbers of discrete behaviors, where selection and adaptation might be presumed to produce the most direct linkage of behaviors to distinct, or encapsulated neuronal pools, modularity was rarely found. For example, the same set of neurons, in the presence of particular neuromodulators, could produce various functional rhythmic behaviors; in marine molluscs with several motor behaviors, single neurons might be engaged in multiple behaviors (Katz, 2011).

Detailed work with *C. elegans* demonstrates that single neurons can participate in generating multiple different behaviors, as a result of the modulation of the neuron's sensitivity, physical connections, and functional connectivity by various chemicals and genes. These findings do not at all discount the existence of functional differentiation between these neurons, but they do suggest that a more nuanced account of their functional complexity is called for. For instance, the olfactory neuron AWCON can direct both attraction and repulsion to the same odor, depending on the presence of specific neuromodulators (Tsunozaki et al., 2008); and the nocioceptive ASH neurons can cause both social aggregation and avoidance, depending on whether the gap junction with RMG neurons and the associated aggregation circuit has been decoupled by the expression of the *npr-1* gene, which encodes a g-protein coupled receptor (Bargmann, 2012). Bargmann writes:

A profound violation of the one neuron-one behavior rule was uncovered by characterizing behaviors under different conditions. For example, avoidance of the repulsive odor octanol at particular concentrations can be generated by two different sets of sensory neurons. In well fed-animals, octanol avoidance is almost entirely mediated by the ASH nocioceptive neurons, but after an hour of starvation, octanol avoidance is distributed between ASH, AWB, and ADL nocioceptive neurons, revealing a change in circuit composition… Food changes the composition of a circuit for oxygen preference behavior (aerotaxis) as well… Aerotaxis is more robust in starved than in well-fed animals, due to the activity of multiple neuromodulators (Cheung et al., 2005; Chang et al., 2006; Rogers et al., 2006)… the *npr-1* neuropeptide receptor that affects aerotaxis also regulates a second behavior, the aggregation of animals into feeding groups. Aggregation is triggered by a number of sensory neurons including the nocioceptive ASH neurons and oxygen sensing URX neurons (de Bono et al., 2002; Coates and de Bono, 2002)… integrated by one pair of npr-1 expressing neurons called RMGs (Macosko et al., 2009)… npr-1 action in RMG uncouples the aggregation circuitry, but leaves the avoidance circuitry intact. This allows ASH to generate different behaviors in two neuromodulatory states. (Bargmann, 2012; pp. 460–461)

By now, the approaches of the descendants of original descriptive electrophysiologists and the neuroethologists have converged, each adding aspects of the initially opposing view, though the intellectual lineages of both can still be traced. Understanding of the complexity of natural scenes and the functions and motivations of perception and behavior came to be part of descriptive neurophysiological studies (Vinje and Gallant, 2000; Brady and Oliva, 2008; Adolphs, 2010). The question of how to embed specific adaptive behaviors in the larger contexts of organization is now being addressed with increasing specificity (Johnson, 2001, 2011).

### Paleontology, anthropology and comparative anatomy

The resolution of the tools physical anthropologists and comparative anatomists are able to use to examine fossil brains, or (historically) could be used to describe primate brains is necessarily crude, but a version of the same local-global, modular- vs. general purpose-circuitry has played out in these fields at a larger scale. The most available measurable entity is whole brain size, allowing taxon-level analysis of changes in the ratio of brain size to body size (“grade shifts”), coupled with analysis of behavioral changes associated with such changes, like homeothermy or carnivory (Jerison, 1973; Northcutt, 1981). Subsequently, primatologists and comparative anatomists attempted to link changes in particular brain parts (e.g., cerebellum; olfactory bulb, a particular cortical gyrus) to changes in behavior (e.g., motor ability, visual vs. olfactory specialists; language), usually after removing shared allometric variation across species. In general, this behavior-to-residual-structural-variation mapping is termed “mosaic” brain evolution. Initially, proponents of mosaic brain evolution pursued the same ends as neuroethologists, in this case attempting to equate species-specific adaptions with the relative sizes of particular brain parts. Overall, initial investigation of mosaic evolution produced rather few interesting generalities, since most behavioral functions are distributed over a number of regions, and allometric covariation of brain parts is extremely high, leaving only a few percentage points of residual anatomical variance to map behavioral variation onto (Stephan et al., 1986; Finlay and Darlington, 1995; Aboitiz, 1996; Finlay et al., 2001; Yopak et al., 2010). For example, in confronting this puzzle, Aboitiz conjectured if there were somehow two different kinds of “size” in the brain, the less-interesting shared variance related to general organismal processes, the remainder important for species-specific adaptations. As a definitional aside here, note that in the case of gross brain morphology, “visual cortex,” for example, will mean anatomically-defined visual cortex to anatomists, and not “regions of brain activated by visual stimulation” as it will, on occasion, for neuroimaging researchers.

A second version of mosaic adaptation emerged, the simple identification of any structural components of variation independent of allometric variation, not necessarily predicted from behavioral specializations (Iwaniuk et al., 2004; Hager et al., 2012), to be considered as potential sources of evolutionary variation and change. Cross-brain-part covariation associated with cognitive and behavioral adaptation of residualized volumes is another way of attempting to locate species-specific adaptations in volume and number differences (de Winter and Oxnard, 2001; Sherwood et al., 2012; Smaers and Soligo, 2013).

For this version of mosaicism or modularity, resolution has not been rapid. Each element of the argument, the nature of the proposed adaptation (e.g., “planning” in humans), the brain part on which the adaptation is to depend (“frontal cortex”), and the survival or reproductive benefit of the adaptation typically remain conjectures. For example, the hypothesis that humans have been specially selected for unusual social competence via specific cortical enlargement, the “social brain hypothesis,” has become quite popular (Dunbar, 1998, 2012). Note that it is the *linkage* of increased volume of particular regions of brain to social ability that is under debate, not whether social structure in humans is unusual. Residual excess cortical size, across all areas (Dunbar, 1993), or enlargement of a particular region involved in the processing of social information (Powell et al., 2012), or perhaps, the presence of a distinctive type of large neuron (Allman et al., 2010) have all been proposed and have not been explicitly resolved by the proponents of this approach. A similar enduring debate is whether the frontal cortex in humans has been the subject of special adaptation in relative size, subregions or neuronal phenotypes, variously associated with aspects of language, multiple behaviors associated with mirror neurons, cognitive control or planning capabilities. Analyses of old and new data for and against this claim have been made over a period of 40 years at this point (of many: Jerison, 1973; Semendeferi et al., 2002, 2011; Schoenemann, 2006; Barton and Venditti, 2013).

By contrast, the behavioral benefits associated with a relatively large brain are of obvious adaptive significance, directly measurable and correlate across taxonomic groups. Simple encephalization across birds, mammals generally, and primates correlates with field measures of behavioral innovation, the rate of success in invasion of new niches, laboratory measures of behavioral flexibility, and reduced mortality in the field (Lefebvre, 2013). Still, a reasonable criticism of the “concerted evolution” interpretation of brain scaling is that large brain divisions like “cerebellum” or frontal cortex” must reflect many of animal's specific behavioral capacities and would certainly contain multiple modules. Thus many important specializations carved out within overall ability might be overlooked. The changes in the neuroethological view of specialization, and the changing views in cognitive neuroscience about functional commitments, however, have come to intersect anthropology and gross comparative anatomy in level of analysis, and they inform each other. That is the reason for considering them together here.

### Cognitive science

Modularity (Fodor, 1983) is a venerable hypothesis in the understanding of the architecture of the mind and brain, arguably dating back to 18th century faculty psychology (Reid, 1785/2002) and its influence on phrenological accounts of brain organization (Gall, 1857). In its Fodorian incarnation, the modularity hypothesis was that (some of) the mind was constituted as a collection of specialized, encapsulated, communicating components—or modules—each dedicated to handling some well-defined aspect of the overall information-processing requirements of the organism. Insofar as this was so, Fodor argued, then each module should have some specific design features. For instance, it should be domain specific, in that it has access to (or at least responds only to) a narrow class of inputs, and transforms these according to some consistent and well-defined function to produce its output; it should be encapsulated, i.e., relatively isolated from influence by the operations of other modules; and it should be implemented in dedicated neural structures. From this perspective, it would also appear that the structures of the brain ought to have some of these same features. Insofar as neural structures are dedicated to particular modules, they will likewise be domain specific and encapsulated with respect to one another. Moreover, each of these design characteristics is mutually supporting in various ways. For instance, a module implemented in neural structures shared with other modules is less likely to be encapsulated relative to those modules; and if a neural structure serves the needs of more than one module, it would appear less likely to be domain specific.

A modular brain, then, would be a collection of domain dedicated, functionally specialized, relatively encapsulated neural structures that together served the information processing needs of the mind. Fodor himself argued that the only parts of the brain likely to be modular “to some interesting extent” (Fodor, 1983; p. 37) were “peripheral” structures dedicated to specialized sensory and motor processing. The probable non-modularity of “central” systems is a result of their hypothesized function of deriving true beliefs, and an argument to the effect that our beliefs are holistically related to one another in various ways—for instance, any belief, regardless of its ostensible domain (e.g., cell biology) could inferentially impact our acceptance of or the consequences we derive from any other belief (e.g., one about summer boat travel in Madagascar). Thus, such central inferential systems are not informationally encapsulated.

Whatever the merits of this Cartesian distinction between peripheral and central systems (cf. Dewey, 1896; pp. 357–358), it seems fair to characterize the current consensus as a rejection of Fodorian modularity as a research guiding idealization of brain architecture. The evidence gathered over the past 30 years overwhelmingly indicates that few parts of the brain or processes of mind appear to have the design characteristics hypothesized by this brand of modularity. Evidence (for instance) for top-down effects on visual processing; for cross-modal integration in perception; for the acute sensitivity of modules to developmental conditions; for cross-modal neural plasticity of many different sorts; and for the implementation of ostensibly distinct processes in overlapping neural structures, including the observation that even very small lesions of the brain typically induce multiple behavioral deficits, all point to a brain organized along rather different principles than those outlined by Fodor (see Barrett and Kurzban, 2006; Prinz, 2006; Anderson, 2010, for reviews).

In response to these critiques, and motivated as well by a desire to integrate psychology and neuroscience more fully with evolutionary biology, advocates of modularity have shifted the focus from the sort of *structurally defined* modularity advocated by Fodor toward a *functionalist* modularity positing a collection of functionally specialized, separately modifiable sub-systems, such that any specific design features of a given module are determined by individual functional requirements not necessarily shared by other modules (Tooby and Cosmides, 1992; Sperber, 2002, 2005; Barrett and Kurzban, 2006; Carruthers, 2006). On this view, sometimes called “massive modularity,” there is no distinction between central and peripheral systems, and the focus is squarely on the evolution of modules that implement solutions to an organism's adaptive problems. This represents a step away from Fodor's Cartesian focus on central belief-fixing representational systems, and toward a more pragmatic, interactive account of the brain's central role in an organism's life. Interestingly, however, massive modularity retains the Fodorian focus on *computation*, and with it a focus on the algorithmic (or heuristic) efficiency of purported psychological solutions to adaptive problems such as food choice, mate selection, kin identification, and cheater detection. The claim is two-fold: that evolution will favor efficient solutions, and that the most efficient solutions will be specialized, domain specific, hence modular components. In many ways this view converges on the approach of the first neuroethologists, though these literatures are virtually independent. Consider the following from Barrett and Kurzban (2006); we quote at length as some of the details of the position will later become important:

Our position, then, is that functionally specialized mechanisms with formally definable informational inputs are characteristic of human (and non-human) cognition and that these features should be identified as the signal properties of “modularity.” By this we intend an explicitly evolutionary reading of the concepts of function and specialization: modules evolved through a process of descent with modification, due to the effects they had on organisms' fitness. … As a direct and inseparable result of this evolutionary process of specialization, modules will become *domain specific*: Because they handle information in specialized ways, they will have specific *input criteria*. … For example, systems specialized for assessing the numerosity of objects accept only representations previously parsed into distinct objects; systems specialized for speech perception process only transduced representations of sound waves; and systems specialized for making good food choices process only representations relevant to the nutritional value of different potential food items. (Barrett and Kurzban, 2006; p. 630)

As should be clear from the quote, it is a central part of massive modularity that each module should be separately modifiable, both in theory, and in the course of evolutionary development [see extensive discussion of this point in Carruthers (2006)]. Indeed, here functional specialization and domain specificity is a *result* of the fact that the modules are separately targeted by evolutionary pressures. Insofar as the focus is on the efficiency of individual computational solutions, as well as the collective efficiency of the system as a whole, a collection of separately modifiable modules that can operate largely in parallel, free of pleiotropy, can easily seem like an elegant design solution, and one to which the various demands of evolution might naturally converge.

And yet, as we have been seeing, the architectures of evolved nervous systems do not seem to reflect this particular solution. So, how should we reconceive the principles governing nervous system evolution? We begin to address this question in the next section.

## From simple to complex, sensorimotor to integrative: mechanisms that control brain size neural plasticity and neural re-use

The large majority of the variation in evolution of vertebrate and mammalian central nervous system numbers can be described as concerted, and allometrically predictable (Stephan et al., 1986; Yopak et al., 2010). Important “grade shifts” in volume allocation often appear at taxonomic boundaries, for example, greater relative volume of the forebrain and cerebellum in mammals compared to reptiles and fish at comparable brain sizes, to which we will return later (Jerison, 1973; Northcutt, 1981; Yopak et al., 2010). In addition, developmental features associated with this conserved evolutionary outcome are being identified: the conserved segmental divisions common to all vertebrate brains (Puelles et al., 2013) coupled with a conserved pattern of neurogenesis whose property of “late equals large” automatically produces disproportionate growth in the same regions (cerebellum, forebrain) in the largest brains across every vertebrate taxonomic group (Finlay et al., 1998). This identification of a developmental mechanism underlying allometric regularity in no way eliminates the requirement that an adaptive account be given of concerted scaling as much as for species-specific adaptation. The notion that a disadvantageous pattern of cell proliferation (appealing to “developmental constraint” as initially hypothesized by Gould, 1977) would be conserved over 450–500 MYA, particularly given the metabolic cost of the brain, is implausible in the extreme. A similar pattern of overwhelming conservation has been described in multiple domains, principally the vertebrate body plan and basic physiological circuits (Gerhart and Kirschner, 1997), and has required the same shift in explanatory style. Given all this conservation, however, species variations in behavior most definitely exist and must be accounted for, considering any collection you choose—catfish, catbirds and cats, for example. The need to explain these profound differences does not disappear if residual variation in brain structure volumes does a poor job in accounting for them. Fresh approaches to this problem will be the focus of this paper.

Considering the adaptive value of conserved scaling, and its niche-independent, brain-size-dependent features, a molar and a molecular account can both be given. The molar account has been discussed elsewhere, and concerns the benefits of this pattern of allometric scaling for a computational device (Finlay et al., 2011; Charvet and Finlay, 2012). Some kinds of computational architectures are simultaneously more amenable to addition or loss of components (such as memory resources) than others (Brooks, 1986; Hawes et al., 2007). Considering the cortex alone, the rostral-to-caudal gradient in length of cortical neurogenesis with its resulting rostral to caudal gradient in increasing neuron number per cortical column, which becomes more pronounced in increasingly larger brains, can be directly related to progressive reduction of dimensions and abstraction of information on that same axis (Charvet et al., 2013).

Here instead we will concentrate on the second problem of how adaptive specializations may be enacted within a generic architecture. First we will look in more detail at the claim that the relative numbers of neurons in or volumes of CNS structures are associated with or are a mechanism of species-specific adaptations. We will particularly underline the idea that many of the demonstrations of such effects, particularly in the case of volumes, may well be describing the downstream effects of the animal's extensive use of a particular sensory modality due to increased elaboration of the sensory periphery, activity changes or motivational state, resculpting the nervous system via its own activity. Changes in brain volumes may often be the result, not the cause of a behavioral change or an alteration in the sensorimotor periphery (Krubitzer and Seelke, 2012). We will also note that the assumption that increase in neuron number should result in improvements in function is often unjustified, particularly when the computational role of each nucleus and neuron class in a functional system is considered.

The potential, empirically well-described, developmental sources of changes in neuron numbers and volume are myriad, even considering only neocortex. These minimally include reassignment of embryonic boundaries (Alfano and Studer, 2013); rate and duration of neurogenesis (Charvet et al., 2011; Workman et al., 2013); respecification of neuronal type or redirection of migration (Letinic and Rakic, 2001); developmental cell death (Finlay and Slattery, 1983; Rehen et al., 2001), and activity-dependent increases in axonal and dendritic arbors produced by experience and resulting changes in cortical volumes (Greenough and Black, 1992; Krubitzer and Seelke, 2012). This list is long if we consider only neuroanatomically defined regions (like “striate cortex”), and even more extensive still if we consider methods, like reuse (Anderson, 2010), by which active inputs may claim processing space in the brain in multimodal or otherwise associative regions. Each developmental mechanism has a range of effect sizes, and developmental onset and offset that should be relevant to our understanding of brain evolution. To understand what kinds of evolution are possible we need to distinguish “primary” genetic changes from the downstream effects of the brain environment of neuroplasticity. Sometimes neuroplasticity might be expected to constrain the effects of undesirable changes, and other times amplify useful ones, and we will supply examples of both.

### Regulation of neuron number in individual brain regions

The idea of specialized, localized functional modules as targets of selection appeared to simplify the problem of selecting for behavioral adaptations by coupling two features both thought to be important in enhancements of brain function. First, special circuitry is often proposed as central to new functionality (from the control of jointed limbs to “grammar modules”). Second, it seems reasonable that more processing resources, neurons and connectivity both, should be committed to important, species-specific capabilities. If both changes could be realized in a single brain part, it would appear to be more efficient than a search of the evolutionary landscape of the entire brain for an optimal combination of dedicated neurons and altered circuitry. As such extreme discrete functional adaptations eventually became to seem unlikely, as discussed earlier under “massive modularity,” a search for how single functional adaptations might be made to “cascade” through spatially separated regions of the developing nervous system was begun. This search produced unexpected results.

The observation that neurons are massively overproduced in early development and die as they establish connectivity (as do synapses) produced a first attempt at neural “evo-devo” (Oppenheim, 1991). The particular case of sexual differentiation of neuron number for sexual behavior in vertebrates is a reasonable entry point, as it involves control of different muscle mass, numbers of motorneurons in the spinal cord and control of the behavior at supraspinal levels. The rat spinal cord begins as uniform in neuron number, and early testosterone allows the survival of the motor neurons associated with the male reproductive apparatus by supporting muscle survival in the periphery; the neurons die in females without the trophic support supplied by the muscle fibers (Lubischer and Arnold, 1995; McCarthy and Arnold, 2011). This hypothesized method of generating system-wide individual differences was eagerly seized as a potential model to sculpt species differences by propagating a single genetic change through the developing nervous system: perhaps a “generic” central nervous system might be generated, and a single specialization, for example, a larger eye, could cascade through multiple sites in the brain by rescuing neurons and synapses from developmental cell death (Finlay, 1992). The functional hypothesis in these series of experiments is similar to the “mosaic” idea of brain evolution: if an animal is specialized for a particular function, there would be benefit for it to amplify the number of neurons committed to that function wholesale throughout its brain.

As initially plausible as this idea might have been, it proved not to be the case, neither for sexual differentiation of individuals nor visual system evolution across species (Oppenheim, 1991; Finlay, 1992). As always, the empirical actuality proved ultimately more interesting than the first guess. Basically, interconnected groups of neurons do not respond with any degree of sensitivity to “match” their relative numbers to each other, but respond with measurable neuron loss only in cases of catastrophic loss of input or target. A single change in neuron numbers at one point in a circuit simply did not propagate past its immediate neighbor. For example, while embryonic complete loss of an eye might cause catastrophic neuron loss in the midbrain and thalamus, and propagate through to change the boundaries of visual cortex, the converse manipulation of introducing large increases in retinal input to the same structures, more relevant to evolutionary adaptations, had little effect, even though there was potentially a great deal of neuron loss and synaptic connectivity the extra tissue might “take up” (Finlay and Pallas, 1989).

The series of experiments of Sarah Pallas and colleagues (Pallas and Finlay, 1989; Huang and Pallas, 2001) on the physiological consequences to visual system organization of numerical imbalances in interconnecting structure provided a case where mechanisms of plasticity appear to work to counter the effects of localized increases in neuron number. These experiments redirected our interest from the idea that increased neuron number in a single structure might be a useful building block of brain evolution to instead, how sensorimotor systems insure the reliability of how they extract information, which we discuss at a little length to illustrate the point. Initially we imagined supplying supranormal retinal input to the midbrain might illuminate how the receptive fields of neurons in the superior colliculus were constructed: we imagined that the receptive field of each cell would have to be twice as large to accommodate the increased input. Nothing of the kind happened: the receptive field sizes of single neurons remained the same. Eventually it appeared that the mapping problem was solved not by reducing cell death in the supra-innervated colliculus, nor allowing increased convergence on single cells, but by increasing the spatial overlap or redundancy of midbrain cells' receptive fields. Activity-dependent mechanisms operating at the midbrain target “permitted” receptive fields only of a certain size, prohibiting plasticity in spatial convergence on single neurons. In hindsight, considering how a functioning visual system should best respond to unexpectedly large ratio variations in neuron number between brain structures, it now seems reasonable that an animal's visual acuity should *never* be dependent on the ratio relationship of its internal parts, but that hard-won peripheral acuity should be maintained over variations as much as possible. In this case, a plastic mechanism works to defend visual function and conserve receptive field properties, and preserve the animal's midbrain-dependent behaviors (Xiong and Finlay, 1996).

When specializations of sensorimotor or behavioral systems in particular species are obvious, what is genetically changed in those species? First, and predominantly, extreme differences can routinely be seen in the sensory and motor periphery. Across sensory systems, the details of peripheral topography will be found faithfully reproduced in the cortex (e.g., Silveira et al., 1989; Catania and Kaas, 1995; Krubitzer and Seelke, 2012; Meyer et al., 2013). An evo-devo account of the minimal number of alterations of neurogenesis to produce the very long list of changes of the eye of the nocturnal owl monkey compared to its diurnal forebears, concentrating on the enrichment of its population of rods and 4 other retinal neuron classes, has been made by one of the authors and her colleagues (Dyer et al., 2009) the point of this example is both to emphasize the multiple specializations for light capture of the nocturnal eye, and the mechanisms coordinating them. Close to the periphery, but definitely within the CNS, special computational devices can be seen, for example, the delay-line neurons of the superior olive that compute time-of-arrival differences for auditory input in the two ears (Carr and Konishi, 1988). Deeper in the CNS, adaptations for special processing that are specified independent of input become progressively more difficult to identify—for example, if there are genetically-specified special transmitters, receptors, or axon lengths in the striate cortex of particular advantage for vision, they have never been explicitly identified. The idea of a “canonical circuit” in the cortex spanning multiple modalities has come to dominate current discussion (Douglas and Martin, 2004; Harris and Mrsic-Flogel, 2013).

While a number of individual reports of selective structural increases of presumed adaptive significance have been made (to be reviewed below), it is worth recalling first that several extensive surveys of particular sensory and behavioral systems with the intention to describe number or volume differences have produced negative results (with the striking exception of the song system in passerine birds). This negative catalogue is rarely cited. For example, Glendenning and Masterton (1998) undertook an analysis of the volumes of 10 subcortical auditory nuclei in 53 diverse mammals, and found they were all highly correlated, and predicted from overall brain size. The three species that deviated most in increased size from the mean values were the little brown bat (other microbats were not unusual), the beaver and the laboratory mouse, the latter two not usually remarked for auditory specialization. Similarly, relative “dexterity,” using a scale ranking animals from hooves to hands, was predicted better from absolute brain size than the relative size of somatosensory cortex (Nudo and Masterton, 1990). Across both birds and mammals, the idea that the relative demands on memory for scatter-hoarding vs. other methods of foraging should be associated with a larger hippocampus enjoyed an initial success (Sherry et al., 1989; Jacobs and Spencer, 1994; Healy and Krebs, 1996), which became progressively less clear as the details of the relationship of real-world foraging to memory, and the lability of hippocampal volume became better understood (Roth et al., 2010; Smulders et al., 2010). A series of studies of the neuron numbers and volumes of visual system structures in primates and mammals including the retina and fovea (Franco et al., 2001; Finlay et al., 2008), lateral geniculate (Finlay et al., 2013), pulvinar (Chalfin et al., 2007), superior colliculus (Cheung, 2003), striate and extrastriate cortex (Kaskan et al., 2005), showed no niche-related effects unexplained by scaling but one (to be discussed) in central structures, major differences of multiple features of the eye and retina, and a substantial “grade shift” between midbrain and forebrain scaling in rodents vs. primates independent of niche. The bird “song system,” however, stands out as a counterexample. Though the identification of the volume of a nucleus specifically with numbers of songs has undergone much elaboration and qualification since its original description (Nottebohm et al., 1981), numerous forebrain specializations have been demonstrated including relationships of neuron numbers to elaborations of capacity, relationship of the same to variation between species (Szekely et al., 1996), and heritability of such differences (Airey et al., 2000). What feature of the song system distinguishes it from the other systems we have reviewed is clearly of major interest. Overall, the point of this catalogue is not to engage the argument that genetically-specified increases in neuron numbers in CNS structures associated with species-specific adaptions have not or cannot ever occur, but that they are simply not as pervasive as an evolutionary mechanism as the list of isolated examples often offered would suggest. In fact, they appear to be rare.

It is worth recalling that while the allometric predictability of neural volumes is very high, the residual variation of individual structures (between and within species) is also high, due to the enormous range of brain sizes.

… The enormous range of structure sizes across species is important for the following reason: In a moderate-sized sample, a normally distributed variable typically has a total sample range of about five times its standard deviation. In predicting the size of brain structures, as noted above, the standard deviation of predictive errors is 0.187 averaged across structures when variables are measured on logarithmic scales. This suggests that for a typical structure, two species identical on our two major factors may have structure sizes differing by as much as 5 X 0.187 or 0.935 on a logarithmic scale. Because exp(O.935) = 2.55, individual structures may differ by a factor of as much as 2.5 in size, even when the two species being compared are very similar on the two major factors. Inspection of the raw data confirms this conclusion. To the investigator seeking evidence for species-specific adaptation, a twofold difference in a structure's volume is striking, even if it is trivial in comparison to the total range of size of that structure and small in comparison to the range of structure size with body size held constant. (Finlay and Darlington, 1995), p. 1580.

So, for example, if a single comparison of the superior colliculus of the nocturnal laboratory rat is made to the diurnal ground squirrel, the two differ in volume by a factor of 10 though their overall brain size is fairly similar (Kaas and Collins, 2001). Unfortunately, however, it is also the case that in the rodent lineage across multiple species there is simply no significant relationship between nocturnal/diurnal niche and midbrain size demonstrable thus far, as laid out in the previous list of citations. Large variations between individuals within a species are often observed, as well. One of the first was the observation of Van Essen and colleagues of nearly four-fold variation in surface area in a small sample of macaque primary visual cortex area (1984). Recently, variation in attention to local vs. global orientation sensitivity has been linked to V1 size (Song et al., 2013) and increased Vernier acuity and decreased susceptibility to two optical illusions (Bakken et al., 2012). An adaptive purpose for these distinctions is not obvious, and no clinically important “small V1 syndrome” has ever emerged. Large ranges in number and volume between individuals within species in at every level of the visual system can be seen, variability characterized by a strong central trend with distinct outliers, but with no identification of the outliers with any obvious behavioral pathology (Franco et al., 2001; Kaskan et al., 2005). It will be interesting to attempt to make specific predictions in any of these cases, but the “rectifying” nature of the neuroplasticity environment discussed earlier for numerical imbalances should be taken into account (Pallas and Finlay, 1989) The case of dramatic “imbalances” in the red/green photoreceptor opsin array unaccompanied by perceptual differences will be discussed shortly (Williams et al., 1993).

Many reported volume increases in single structures linked to niche-specific variations are likely to be the outcome of a “generic” nervous system operating in an unusual niche: the effects of environmental deprivation and enrichment on primary visual cortex volumes, for example, range around 5–10% (Greenough and Black, 1992), and feral rats differ from laboratory rats in these approximate magnitudes (Krubitzer et al., 2011). Not all observations can be described this way: two species of squirrels appeared to have larger relative areas of visual compared to somatosensory cortex, which will be useful for further investigation of phylogenetic vs. developmental causes of this kind of specialization (Krubitzer and Seelke, 2012). Other examples of “coordinated” changes within functional systems seem likely to be developmental, if not tautological in origin: synaptically connected structures within functional systems literally contain volumetric components of each other in their axonal inputs (Barton and Harvey, 2000; Barton et al., 2003).

In the study of primate vision, contesting and conflicting accounts of the adaptive purpose of visual system features are ceaselessly argued. For example, considering the trichromacy of New and Old World primates [whose species comprise a majority of frugivores, but also insectivores, folivores, carnivores, tree-gum-specialists and omnivores like ourselves (Fleagle, 1999)], convincing evidence that trichromacy improves scene segmentation (Hansen and Gegenfurtner, 2009), distinction of shading from reflectance (Kingdom, 2003), social communication involving detection of blood oxygenation, (Changizi et al., 2006), foraging for fruits (Regan et al., 2001), or distinction of leaf age for folivory (Lucas et al., 1997) have all been offered; clearly it is likely that trichromacy contributes to all of them. But when we leave psychophysics and turn to the brain, details of what adaptation and what processing model best describe the primate visual system are left behind, using the assumption of “more is better” for every stage of visual system organization. For example, Barton argues that a statistically demonstrable increase in the P/M cell ratio of the lateral geniculate is a special adaptation for frugivory in diurnal primates. The ratio of parvocellular neurons (P cells, small cells, primarily representing the fovea, specialized for high spatial acuity and one aspect of trichromacy) vs. magnocellular neurons (M cells; larger neurons, more evenly spread across the retina; higher temporal acuity and participating in dichromacy only) in the lateral geniculate increases with regular allometry with brain size and is slightly higher in diurnal primates than nocturnal ones (Barton, 1998, 2004). We were recently able to confirm and extend this observation (P/M ratio higher in diurnal primates) to more primate species (Finlay et al., 2013), but also show that the cause was likely to be the developmental cell death of the M neurons representing the visual periphery, competing unsuccessfully for synaptic space in primary visual cortex dominated by early arrival and topographic occupation of the cortex by the foveal representation, a hitherto unexplained developmental observation of Williams and Rakic (1988). The cause of the changed ratio, therefore, is not a genetic change directly producing greater P cell numbers, but a downstream effect of the developmentally “generic” mechanism of active and early-generated sensory specializations claiming greater synaptic space.

The differing computational role of number at different stages of sensory and cognitive analysis needs to be considered as well when considering allometric relationships—how much more is better? For example, in the primate retina, cones, because they are flooded with photons in high light levels, may sample a large visual angle selectively without loss in acuity without increase in number as the eye enlarges, while rods normally carpet the retina to maximize sensitivity. The allometric outcome is that rods increase rapidly in number with eye size while cone numbers change little, so that humans, the primate with the largest eye, have by far the most rods of the diurnal primates (Finlay et al., 2008). The optic nerve and primary visual thalamic nuclei, in concert with sensory thalamic nuclei in general, appear to be defended as a bottleneck, and do not increase rapidly with brain size. This phenomenon has several interesting candidate functions, such as production of an efficient compression or multiplexing of retinal input, or roughly equilibrating the information contributed by various sensory modalities to the cortex (Fetsch et al., 2013).

Overall, we argue, with the several empirical exceptions noted, that the evidence for structure-by-structure genetic selection on neuron number for particular adaptive ends is surprisingly poor, especially given the pervasive belief the phenomenon should exist. Most of the variance in neuron number is shared, and the simple existence of unshared variance by itself is not evidence for “mosaic” evolution. Marked individual local variations in number so far have not advertised their functional consequences, and in at least a few cases, appeared to be actively compensated rather than exploited. Within the visual system, and perhaps for sensory systems generally, a niche-independent computational role for the scaling of each cell group can often be identified. Several striking time points in vertebrate evolution exist where changes relative proliferation of brain parts accompanies a niche change, such as those associated with homeothermy, or becoming terrestrial. Interestingly, though, in those cases, the structures with the highest allometric slopes in the stem group are the same ones that are further amplified in the derived group. There is, however, at least one distributed, covarying and relatively independent system that can be discriminated within the brain, from the onset of vertebrate evolution. This is the “limbic” system, comprising olfactory bulb and cortices, hippocampus and various forebrain nuclei, which varies relatively independently from the rest of the brain (Jerison, 1973; Finlay and Darlington, 1995; Reep et al., 2007). Why this relative independence should have persisted for 450 MY, in tandem with the regular scaling of the rest of the brain, is a very intriguing question. Several distinctions can be made between these functions of these large systems, the first olfactory vs. visual specialization, but that does not exhaust all possibilities. Egocentric vs. allocentric spatial representations, and short-term vs. very long term memory storage distinguish these systems as well.

### Examples of multifunctionality in nervous systems, from neurons to regions

In light of such increasingly common discoveries as those detailed above, a different perspective on functional brain evolution and organization has begun to emerge, that puts the focus not on the selective targeting of individual structures, but instead on overall efficiency in deployment of neural resources. According to these so-called neural reuse theories (Anderson, 2010), resource constraints and efficiency considerations dictate that, rather than developing new structures *de novo*, whenever possible neural, behavioral and environmental resources should have been reused and redeployed in support of any newly emerging cognitive capacities. That is, rather than following an evolutionary/developmental pathway wherein organisms develop specialized, dedicated neural hardware to meet each new adaptive challenge, reuse suggests that much local neural structure is conserved but is often combined and recombined by different organisms in different ways to achieve diverse purposes. The fact of functional *differentiation* between parts of the brain need not imply the existence of functional *specialization* in all such cases.

We have, of course, already seen many examples, and such reuse of neural elements to regulate multiple behaviors seems to be the rule rather than the exception in the nervous systems of many animals. Examples of neural reuse can be found across the animal kingdom, suggesting it is a vitally important evolutionary strategy for deploying scarce neural resources to the greatest behavioral and adaptive effect.

Reuse may also be found in neurons involved in learning and memory. In the pond snail (*Lymnea stagnalis*), the breathing rhythm is generated by three synaptically connected neurons that form a central pattern generator. One of these neurons, RPeD1, is also necessary for many aspects of learning and memory; and removing the RPeD1 cell body can prevent the formation or reconsolidation of long-term memories (Sangha et al., 2003). In honeybees (*Apis mellifera*), a single identified neuron (VUMmx1) in the suboesophageal ganglion mediates the reward pathway in associative olfactory learning, but this neuron has also been implicated in learning phenomena as diverse as second-order conditioning and blocking (Menzel, 2009). (Niven and Chittka, 2010; p. 285)

Similar neuromodulation comes in many guises in vertebrates. A textbook example of neural re-use employing gain and gating changes is the “duplex” retina: beginning from the receptors, the neurons of the retina can be engaged, de-coupled, or have their processing features entirely reorganized depending on whether they are participating in scotopic, (low light or nocturnal) vision, or photopic, (high light level or diurnal) vision (Palmer, 1998). Vertebrate vision appears to have originally arisen for conditions of high light levels, and adaptations for higher sensitivity in dim light appeared secondarily, employing the same retinal neurons (for a general review, Bowmaker, 2012). Transitions in visual niche have occurred repeatedly, within large taxonomic groups, such as those including sharks and rays (Yopak et al., 2010), at the emergence of the first mammals occupying nocturnal niches, and notably in primates, where diurnal primates emerged from primarily nocturnal stem species (Ross, 2000; Gerkema et al., 2013). At the cellular level, the catalogue of adjustments is long, but one example is illustrative: for low-light vision, the lateral inhibition opposing the responses of the center and periphery of visual receptive field normally seen in diurnal vision is removed, increasing sensitivity and reducing spatial acuity. This alteration can be produced directly, by a change in ambient light level, or predictively, in accord with circadian rhythmicity (Palmer, 1998). Although the nearest-neighbor relationship of retinal cells is preserved in low-light vision, the central specialization of the fovea is essentially removed as well. The basis of receptive field structure in diurnal vision, lateral inhibition, is removed from retina, lateral geniculate and visual cortex in nocturnal vision, yet the same structures are used to see.

The transition from night to day vision in individuals in species that can function in both milieus, notably ourselves, is easy. Interestingly, the wholesale evolutionary transition from a retina adapted to diurnal vision to nocturnal vision (in New World monkeys, Dyer et al., 2009) is similarly easy, harnessing the temporal relationships of neurogenesis seen in the diurnal peripheral retina to change the complement of all types of retinal neurons in one step. The availability of re-use in central nervous system targets of the retina allows this complex transition to be produced by changing timing relations in a few control steps in the retina alone, rather than by respecifying the physiology of each and every participating neuron directly, or worse yet, having to generate a second nocturnal eye.

Finally, there is a good deal of emerging work that points to the importance of the large-scale modulation of neural partnerships in support of cognitive function. For instance, changes in the oscillatory coherence between brain regions (local and long-distance) appear to be important to sensory binding, the modulation of attention, and other cognitive functions (Steinmetz et al., 2000; Uhlhaas et al., 2009; Varela et al., 2001; Fries, 2009; Nacher et al., 2013). The basic finding that cognitive function involves the reuse of the same elements in different configurations is illustrated by two early studies: Friston (1997) demonstrated that whether a given region of inferotemporal cortex was face selective depended on the level of activity in posterior parietal cortex; and McIntosh et al. (1994) report on a region of inferotemporal cortex and a region of prefrontal cortex that both support face identification and spatial attention. In the latter study, McIntosh and colleagues showed that during the face processing task the inferotemporal region cooperated strongly with a region of superior parietal cortex; while during the *attention* task, that same region of parietal cortex cooperated more strongly with the prefrontal area. Similar patterns of changing functional connectivity are observed over developmental time, which suggests that acquiring new skills involves changes to both local and long-distance functional partnerships (Fair et al., 2007, 2009; Supekar et al., 2009).

Our examples, due to the specializations of the authors, are principally drawn from vision and human cognition, but it is worth noting that it presents little challenge to find comparable examples in motivational and emotional domains. In voles, the transition from principally promiscuous to principally monogamous mating systems, both between species, between individuals, and perhaps over development, is thought to involve the interposition of a vasotocin or oxytocin receptor gate involving individual recognition in basic reinforcement circuitry (Insel and Young, 2001). Adjustment of sensory gain can be seen in the stress-induced analgesia observed in both rodents and humans (Akil et al., 1984; Bargmann, 2012). Stress can also change the configuration of large scale brain networks across a number of species including humans (Hermans et al., 2011), including early-stage sensory processing depending on emotional arousal, as demonstrated in V1 by Mourao-Miranda et al. (2003).

Some intriguing further evidence for the reuse of larger neural elements comes from data-mining large collections of human neuroimaging studies. For example, Poldrack (2006) estimated the selectivity of Broca's area by performing a Bayesian analysis of 3222 imaging studies from the BrainMap database (Laird et al., 2005). He concludes that current evidence for the notion that Broca's area is a “language” region is fairly weak, in part because it was more frequently activated by non-language tasks than by language-related ones. Similarly, several whole-brain statistical analyses of large collections experiments from BrainMap (Laird et al., 2005), Neurosynth (Yarkoni et al., 2011) and other sources demonstrate that most regions of the brain—even fairly small regions—appear to be activated by multiple tasks across diverse task categories (Anderson, 2010; Anderson and Penner-Wilger, 2013; Anderson et al., 2013).

The observable large-scale patterns of use and reuse of individual regions of the brain across multiple circumstances suggests that this functional diversity is a reflection of the evolutionary and developmental history of the human brain. For instance, it appears that, *ceteris paribus*, the “older” regions of the human cortex, the primary sensory areas possessed by every mammal (Krubitzer, 2009) as distinguished from the various association regions which appear selectively in larger brains, tend to be used in more tasks—presumably because they've been around for longer, and have thus had more opportunity to be incorporated into multiple functional coalitions (Anderson, 2007). In addition, more recently emerging cognitive functions, such as language, appear to be supported by more and more widely scattered brain regions than are evolutionarily older functions such as vision and attention (Anderson, 2010; Anderson and Penner-Wilger, 2013). Again, this makes sense in light of both progressive functional differentiation and evolutionary continuity, for the later a given cognitive process or behavioral competence emerges, the greater the number and diversity of neural structures that will be available to support the new competence, and there is little reason to believe the useful structures will be near one another in the brain.

Thus, while massive modularity and neural reuse both agree that functional brain architecture needs to be understood in an evolutionary framework, these positions differ on the question of where and how evolutionary pressures are likely to be felt. In particular, it is important to notice the following crucial implication of widespread neural reuse for massive modularity: insofar as these different cognitive and behavioral capacities are supported by reusing many of the same neural elements in different functional coalitions, it is hard to see how it would be possible to separately target and modify these coalitions via natural selection [or by any other means; for further discussion see (Anderson, 2010, 2014)]. Functional “modules” that are built out of shared parts will rarely be separately modifiable; these findings thus encourage a shift in thinking away from massive modularity and individually tailored and inherited solutions to adaptive problems, and toward models that favor more concerted evolution.

### Functional differentiation within a generic nervous system: three evo-devo solutions

All of the foregoing does raise a crucial question: how can one get specialized, differential, heritable function in nervous systems where concerted evolution appears necessary for both functional and architectural reasons? The key is seeing how evolutionary and developmental mechanisms can work together. We will describe three evo-devo interactions for which evidence exists. The first is a canonical example of “evolvability” in which existing information processing mechanisms accept and immediately employ a new dimension of sensory information when it is made available by a genetic change in the sensory periphery. The second example explores the interaction between motivational “presets” and the population of the central nervous system with the information the organism then preferentially acquires. Finally, we will discuss mechanisms of active search for available neural resources.

For the first example, cooperation between evolutionary and developmental mechanisms can be seen in the case of color vision plasticity (Neitz et al., 2002). A remarkable fact about color vision is that there is very little inter-individual variation in performance, despite immense differences in the (largely) genetically specified ratio of L to M cones in the retina. There is, for instance, almost zero variation in the wavelength of light judged to be “uniquely yellow” (without red or green tint), despite a 25 fold variation in LM cone ratio (Williams et al., 1993).

In a series of experiments, (Neitz et al., 2002) systematically altered the color environments of several adult subjects through the use of colored contacts, special lighting schemes and similar measures. They showed that there exists a cortical mechanism for adjusting the sensory gain, such that the signal received from the L and M cones remains in equilibrium under prevailing environmental conditions—that is, given the mean chromaticity of the experienced environment. They hypothesize that this developmental mechanism allows for standardized color vision despite genetic and environmental variation. The selective advantage of such standardized color vision would tend to stabilize the evolutionary and developmental mechanisms that produce it. They hypothesize that this is the identical mechanism that permits the trichromacy that normally arises by the mutation of one opsin in about two thirds of female New World monkeys in the absence of any known brain changes (Jacobs, 2012), and the rapid emergence of behavioral trichromacy after “knock-in” of a third opsin into normally dichromatic monkeys (Mancuso et al., 2009).

For our second evo-devo example, the motivational “presets” which certainly vary between species and may vary between individuals, will certainly alter which environments individuals select, what sensory stimulation they seek, and thus how their brain, and particularly the cortex, becomes populated with information. For example, some species of birds are solitary, and are made anxious or aggressive by the presence of conspecifics, while others have the opposite response, related to non-apeptide distribution in the basal forebrain (Goodson et al., 2012). The progeny of these birds, whether or not the individual offspring itself has the corresponding motivational bias, will grow up in an environment absent of most other birds in the first case, and full of birds in the second. The most well-known example in the human literature of such a “preset” is the preference of infants to look at the human face. Human infants prefer to look at face-like configurations (Johnson, 2011), and will work hard and learn quickly for social reinforcement (Goldstein and Schwade, 2008). Even with the initial preference for faces, being likely subcortical (Johnson, 2001), the advantages of the eye coloration and our contrasting sclera enabling gaze-tracking, and enthusiasm for social learning, it still takes 7–10 years for the representation of faces in the cortex of human children to begin to approximate adult organization (Cohen Kadosh et al., 2011). In autism, abnormal social interaction, abnormalities in both early and late patterns of eye movements, and alteration of face-processing regions in the cortex all co-occur (Kennedy and Adolphs, 2012). The predisposing condition to produce a cortex with a substantial percentage of its volume involved in processing faces and the nuances of emotional expression might only need motivated coupling of early eye and head movements toward expressive faces over long developmental time. The question of how this information is allocated to regions is a question that can be studied phylogenetically, developmentally, and in the service of individual differences.

Finally we consider active search for coordinated input. In addition to instances of sensory gain modulation involving the tuning of local neural responses, there is some suggestive evidence for a neural “search” mechanism that works to establish functional partnerships between cells and between cortical regions. For instance, learning to control an artificial limb via a direct cortical interface (a so-called brain-machine interface, or BMI) appears to involve both an alteration of the tuning curves for individual cells, and also a change in the patterns of functional correlation between cells in the local network. Lebedev and Nicoleilis (2006) describe the neural effects of the learning process this way:

[C]ontinuous BMI operations in primates lead to physiological changes in neuronal tuning, which include changes in preferred direction and direction tuning strength of neurons (Taylor et al., 2002; Carmena et al., 2003; Lebedev et al., 2005). In addition, broad changes in pair-wise neuronal correlation can be detected after BMIs are switched to operate fully under brain-control mode (Carmena et al., 2003; Lebedev et al., 2005).

Along with these physiological adaptations of neuronal firing patterns, behavioral performance improves as animals learn to operate BMIs effectively (Taylor et al., 2002; Carmena et al., 2003; Lebedev et al., 2005). Initial training to operate a BMI is characterized by an increase in neuronal firing rate variance, which cannot be simply explained by changes in limb or actuator movements (Zacksenhouse et al., 2005). As the quality of BMI control improves, initial elevation of neuronal firing variability subsides. (Lebedev and Nicoleilis, 2006: 542)

In so far as oscillatory coherence between cells is a sign of functional cooperation, then it is intriguing to note that one effect of the observed increase in neuronal firing rate variance is to implement a walk through cellular coherence space. That is, as the firing rates of the cells change, they will come into synchrony with a series of different partners over time. The suggestion is that the partnerships that produce the desired effects will be strengthened, with the end result being the establishment of a set of neural partnerships (via “broad changes in pair-wise neuronal correlation”) able to control the limb.

Similar search mechanisms may be behind cases of sensory substitution, in which input from one sensory modality (e.g., touch) is used to provide information normally provided by another (e.g., vision), as with the use of a prosthetic camera that transmits information via mechanical or electrical stimulation to the skin (Bach-y-Rita et al., 1969), an effect generated more prosaically when using Braille dots for reading. As is by now quite well known, in such cases parts of the brain that would normally support processing of information in the original sensory modality can come to support the processing of input in the new modality (Pascual-Leone and Hamilton, 2001; Merabet et al., 2004). Thus can occipital cortex, normally associated with visual processing come to support tactile processing in these and other cases (Zangaladze et al., 1999; Amedi et al., 2002; Merabet et al., 2004; Pietrini et al., 2004). In one particularly interesting case, Merabet et al. (2008) taught sighted individuals to read Braille while blindfolded, and verified using both fMRI and rTMS that occipital cortex was part of the supporting network for the skill. However, after removing the blindfolds, participants remained able to read Braille but no longer showed activation in occipital cortex. The skill was now “presumably supported by activity at brain regions other than the occipital cortex.” (Merabet et al., 2008; p. 8)

Together, these and other pieces of evidence suggest that the mechanisms underlying neurofunctional development (early as well as late skill acquisition) include a process of active search: the rapid testing of multiple neural partnerships to identify functionally adequate options, in some cases multiple alternative possibilities, leading to a degenerate functional network that can be modulated depending on circumstances and task demands (Sporns, 2011; Bargmann, 2012; see Anderson, 2014 for extensive discussion).

Although the foregoing can hardly be said to establish this fact, if granted the assumption that the brain possesses mechanisms for functional development that include both the ability to tune local neural structure in response to task-relevant statistical properties in inputs, and the ability to perform a “search” for functional partnerships between structural elements at various spatial scales, then it becomes possible to see how systematic, heritable, and relatively consistent functional differentiation in the brain could occur in the absence of targeted modular or mosaic selection. Given a set of early developing and stereotyped neural projections from sensory afferents, and assuming an environment that is largely conserved over generations, local tuning mechanisms would be sufficient to produce local networks with specific and predictable functional structures and response tendencies. A set of such neural structures with different functional biases (different input-output mappings) would be enough to allow an ongoing process of neural search to identify and consolidate the sets of partnerships that reliably supported skills being acquired during development. Consistency in the early development of functional biases and in the nature of the tasks being learned by the organism would be sufficient on this model to produce relatively consistent large-scale functional networks, without the need for direct evolutionary targeting. Indeed, in a functionally differentiated but non-modular brain, selection pressures might work not to produce particular specializations, but rather to stabilize the availability of a diverse mixture of computational properties in the entire brain (Atallah et al., 2004) coupled with a range of cortical biases that, given sensory inputs and the interactions between regions (Johnson, 2001, 2011) reliably produces the functional architecture we observe.

## Summary and conclusions

So, then, how do we resolve the apparent paradox that existing developmental processes must have been selected both to be robust to perturbations and accidents, but not so robust as to be unevolvable? The key lies in seeing how the nature of evolutionary adaptations of the brain and developmental mechanisms are intertwined and mutually supporting. For instance, insofar as the properties of environments are generally conserved between generations, then developmental mechanisms that are sensitive to those properties will reliably produce “heritable” specializations, while still being able to compensate when environments change. Here it is worth emphasizing as well the various ways in which the behavior of organisms themselves serves to change and stabilize the environment through various kinds and degrees of niche construction (Odling-Smee et al., 2003; Richerson and Boyd, 2005). When this is combined with the insight that the brain has a meta-modal, domain general organization (Pascual-Leone and Hamilton, 2001; Anderson, 2010), it becomes possible to see how brain architecture can be robust to perturbations and reliably produce a diverse range of different specialized processing operators whose cooperation can support species-typical behaviors. When either opportunity or disaster strikes, the absence of genetically-specified, domain-specific, stereotyped, modular structures and the multiple mechanisms of neuroplasticity permit evolvability.

### Conflict of interest statement

The authors declare that the research was conducted in the absence of any commercial or financial relationships that could be construed as a potential conflict of interest.
